# Prevalence of overweight and obesity and associated factors in school children and adolescents in a medium-sized Brazilian city

**DOI:** 10.6061/clinics/2018/e438

**Published:** 2018-11-16

**Authors:** Adriana Paula da Silva, Taciana Carla Maia Feilbelmann, Daniela Cristina Silva, Heloisa Marcelina Cunha Palhares, Lúcia Marina Scatena, Elisabete Aparecida Mantovani Rodrigues de Resende, Maria de Fátima Borges

**Affiliations:** I Divisao de Endocrinologia, Universidade Federal do Triangulo Mineiro, Uberaba, MG, BR; II Departamento de Medicina Social, Universidade Federal do Triangulo Mineiro, Uberaba, MG, BR

**Keywords:** Children, Adolescents, Overweight, Obesity, Nutritional Status

## Abstract

**OBJECTIVES:**

The objective of this study was to describe the prevalence of overweight and obesity in school children and adolescents in a medium-sized Brazilian city.

**METHODS:**

In total, 1,125 children and adolescents between the ages of 5.6 and 18 years from public and private schools participated in the study. The sample included 681 girls and 444 boys. Each subject's weight and height were obtained according to Brazilian guidelines (SISVAN). The triceps (TSF), subscapular (SSF), biceps, suprailiac, femoral and calf skinfolds were measured in triplicate. Body mass index (BMI) was classified as the BMI percentile (BMIP) according to the World Health Organization (WHO) 2007 criteria. The percentage body fat (%BF) was obtained using the equations by Slaughter et al., 1998. Categorical variables were analyzed using the chi-squared test.

**RESULTS:**

Overall, 364 participants with excess weight were identified: 17.3% were overweight, and 15.0% were obese. Among the girls, 18.0% were overweight, and 12.5% were obese; among the boys, 15.3% were overweight, and 18.0% were obese. These prevalence rates were higher when the time spent watching TV or participating in media-related activities surpassed 5 hrs/day, when individuals belonged to a higher economic class and when the head of the family had a higher education level (≥12 years).

**CONCLUSION:**

It is important to emphasize the need to increase our understanding of factors associated with overweight and obesity, and it is essential to implement measures and policies aimed at reversing this trend, such as stimulating healthy eating habits and physical activity and reducing time spent watching TV and participating in other media activities, including video games and social networking.

## INTRODUCTION

Obesity is defined as an excess of body fat mass compared to a lean body mass, resulting in a positive energy balance 1. Obesity has increased both in adult populations and among children and adolescents 2, it affects both developed and developing countries 3, and it has been associated with the development of cardiovascular disease (CVD), hypertension, metabolic syndrome, some types of cancer (CA), joint impairments, psychological repercussions, and a decreased quality of life 4.

According to the World Health Organization (WHO), in 2008, 35% of adults over 20 years of age were overweight, and 11% were obese. In 2011, approximately 40 million children under the age of 5 years were classified as overweight 5. In the United States in 2009-2010, the National Center of Health Statistics (NCHS) reported obesity prevalence rates of 18.6% and 15% among male and female adolescents, respectively 6.

In Brazil, the 2008-2009 Household Budget Survey (Pesquisa de Orçamentos Familiares - POF - 2008-2009) analyzed the secular trend of anthropometric indicators in the 5- to 9-year-old and 10- to 19-year-old age groups and observed dramatic increases in the prevalence rates of overweight and obesity over a 34-year period 7. Recently, a large Brazilian study (the ERICA study) analyzed 73,399 adolescents and reported national overweight and obesity prevalence rates of 17.4% of 8.6%, respectively—similar to those in the southeastern region of the country of 17.1% and 8.4%, respectively. Even though Minas Gerais is in the southeastern part of Brazil, the ERICA study 8 did not involve any cities in the Triângulo Mineiro region, which has peculiar demographic and socioeconomic characteristics.

The current study aims to fill this gap and intends to report the prevalence of overweight and obesity in the municipality of Uberaba, a medium-sized city in the Triângulo Mineiro in the state of Minas Gerais (MG), Brazil.

## MATERIALS AND METHODS

This cross-sectional study was carried out from March 2012 to August 2013 and included male and female children aged 5-18 years old from public and private schools. The project was approved by the Human Research Ethics Committee of the Federal University of Triângulo Mineiro (Universidade Federal do Triângulo Mineiro – UFTM) under Protocol No. 848/2007.

The study cohort was chosen from a population of 48,390 (according to the 2009 census) primary and secondary children enrolled in public and private schools in Uberaba. The sample size calculation was performed in 3 steps 9. First, a simple random sampling of the population was performed. The second step defined a proportional share of the sampling based on the distribution of students enrolled in public and private schools. A total of 889 students (85%) attended public schools, and 157 (15%) attended private schools. The third step was achieved through a conglomerate sampling technique to establish the numbers of public and private schools (sample units) to be sampled to obtain the previous sample size calculation 9,10.

In total, 15 public and 5 private schools were randomly selected, as public schools did not include all educations levels. To account for the possible loss and non-acceptance of students, the sample was tripled. Thus, 180 participants from each public school and 100 from each private school were randomly selected.

Permission to conduct the study was then requested from each school's administration after providing them information on the study's aims and methodologies. Parents or legal guardians of the children and adolescents were invited to visit the school at a specific date and time. A semi-structured questionnaire and an informed consent form were given to the parents, who were instructed on how to complete it. They were then given one week to fill out the questionnaire and sign the consent form before all of the materials were collected. Contact was made by telephone, and/or the presence of a parent/guardian was requested at the school to finish incomplete questionnaires. After 2 unsuccessful attempts to collect the materials, the student was excluded from the study.

Trained staff consisting of 5 nutritionists and a medical endocrinologist performed the physical examinations. The children's weights were measured using a digital electronic scale (BALGL3C model manufactured by G-TECH, China) with a capacity of 180 kg and accuracy of 50 g; the children's outer clothing and shoes were removed for this measurement. Their heights were determined using the Altura Exata^®^ stadiometer (Belo Horizonte, MG, Brazil), which measures up to 213 cm and has divisions in millimeters. For this measurement, participants stood upright and erect with their feet slightly apart and their heels touching the base of the appliance 11.

An adipometer (Cescorf Cient�fico, Porto Alegre, RS, Brazil) was used to measure skinfold thickness 12,13. Waist, abdominal and hip circumferences (HCs) were measured in centimeters using an inextensible measuring tape (Cescorf^®^) 13. To define the nutritional statuses, the following criteria were used: malnourished participants had body mass index percentiles (BMIPs) of less than P3 (<P3), eutrophic participants had BMIPs equal to or greater than P3 but less than P85 (≥P3<P85), overweight participants had BMIPs equal to or greater than P85 but less than P97 (≥P85<P97), and obese participants had BMIPs greater than or equal to P97 (≥P97) 11.

The percent body fat (%BF) was calculated from the measurements of the triceps skinfold thickness (TSF) and the subscapularis skinfold (SSF) thickness using the equations developed by Slaughter et al. 12,14. The conversion to %BF was made according to the previous work of Deurenberg et al. 15.

The time spent watching TV, playing video games or using a computer (denominated sedentary leisure activities) was reported on the semi-structured questionnaire. The study participants, along with their parents or guardians, completed the pubertal development assessment 9. They specified their current stage via photos representing the various pubertal phases proposed by Marshal and Tanner 16 and adapted by Chipkevitch 17.

The socioeconomic level was established in accordance with the Brazilian Economic Criteria from the Brazilian Association of Research Companies (ABEP – *Associação Brasileira de Empresas de Pesquisa)* 18. The classes defined were upper class, middle class and lower class, as suggested by the Department of Strategic Affairs (SAE – *Secretaria de Assuntos Estratégicos*) of the Presidency of the Republic of Brazil 19. For stratification by ethnicity, we used the self-reported skin color as described previously by other authors 9,20.

Data were organized in a Microsoft Excel^®^ spreadsheet for subsequent analysis. A description of the variables was also included on the spreadsheet. The chi-squared test (*χ*^2^) was used to compare the categorical variables. The data were analyzed using the Statistical Package for the Social Sciences (SPSS) version 20.0 (SPSS Inc., Chicago, IL, USA).

## RESULTS

In total, 1,125 children between 5.6 and 18 years of age, including 681 (60.5%) female and 444 (39.5%) male students, were evaluated. A total of 727 (64.6%) students were classified as eutrophic, while 398 were considered to have altered nutritional statuses, including 34 (3.0%) malnourished, 195 (17.3%) overweight and 169 (15.0%) obese. The prevalence rates of overweight and obesity grouped by demographic and socioeconomic variables and body composition are shown in Table 1.

Overweight was more prevalent in females (n=127, 59.9%), and obesity was more prevalent in males (n=84, 55.3%) (*p*=0.004). There were no significant differences between the prevalence rates of overweight and obesity according to skin color. Participants classified as post-pubertal had a higher prevalence of overweight (n=73, 63.5%) than obesity (n=42, 36.5%) (*p*=0.036). There were no significant differences in socioeconomic status, education level of the head of the family or administrative category of the schools in relation to the total prevalence rates of overweight and obesity.

The prevalence rates of overweight and obesity grouped according to sex and demographic/socioeconomic variables are shown in Table 2. When grouped according to skin color, among white children, obesity was more common in males (n=40, 62.5%), while overweight was more common in females (n=53, 55.7%) (*p*=0.035).

The influence of puberty was assessed by dividing individuals according to sex and pubertal stage. The analysis showed that obesity (n=45; 69.2%) and overweight (n=33; 52.4%) (*p*=0.002) were more common in prepubertal male participants, while in the pubertal individuals, overweight (n=44; 74.6%) and obesity (n=40, 64.5%) (*p*<0.0001) were more common in female participants.

Participants from families whose heads of family had ≥12 years of education had higher prevalence rates of overweight in females (n=70, 61.9%) and obesity in males (n=48, 57.2%) (*p*=0.006).

In public schools, overweight was more common in females (n=76, 66.7%, *p*<0.0001), and obesity was more common in males (n=49, 48.4%, *p*<0.0001). When considering economic class, among those in the highest economic classes (A + B), overweight was more common in females (n=61, 58.6%, *p*=0.016), and obesity was more common in males (n=44, 58.7%, *p*=0.016).

When the time spent on sedentary leisure activities was ≥5 hrs/day, there was increased prevalence rates of overweight among females (n=47, 66.2%, *p*=0.027) and obesity among males (n=27, 54%, *p*=0.027).

The correlations between %BF and sedentary activity in hours/week and between %BF and physical activity in minutes/week were grouped according to sex. There was a statistically significant and moderate correlation between %BF and sedentary activity in the overweight group in both sexes, but no significant correlations were observed between %BF and physical activity in either group or sex, as shown in Table 3.

## DISCUSSION

The prevalence rates of overweight and obesity found in our study is yet another cause for concern for the health of children and adolescents in Brazil. The percentages observed here—17.3% overweight (18.0% of females and 15.3% of males) and 15.0% obese (12.5% of females and 18.9% of males)—were similar to those reported by Dumith and Farias Júnior 21. In their study, conducted in Rio Grande in the state of Rio Grande do Sul (RS), the authors described and compared the nutritional statuses of 525 school children between the ages of 7 and 15 years using 3 BMI-based criteria. The prevalence of excess weight (overweight and obesity) was 35.1%, which is similar to the rate found in our study (32.3%). In a study that included students from public and private schools in Ouro Preto (MG) 22, 20.1% of students exhibited excess weight; however, the authors did not differentiate between the percentages of overweight and obesity.

Comparing our data from the adolescent group with that from the ERICA study conducted in 2014 in MG 8, we observed a similar prevalence of overweight (17.1% vs. 17.3%), but surprisingly, the prevalence of obesity in our study was higher (8.4% vs. 15%) and was even doubled in males (18.9%). This finding could be attributed to regional characteristics of the different populations.

In fact, analysis of the ERICA study 8 revealed that it involved Belo Horizonte and at least 6 cities near the capital, which provides only a limited picture of the true situation. MG has 850 municipalities and is divided into 12 mesoregions that have geographic and socioeconomic differences.

Uberaba, which was analyzed here, represents a medium-sized city located in the mesoregion of Triângulo Mineiro in MG that is strategically equidistant, within a 500 km radius, from the main urban centers of the Southeast and Central-West regions. It has a well-developed economy in 3 major sectors (agriculture, industry and trade) and the 4th largest human development index in MG. It also has good quality-of-life indices. In 2013, the number of students at all education levels totaled 112,000 23. With this profile, this municipality is positioned as an important socioeconomic development center in the country; however, it has many of the same challenges as other medium-sized Brazilian cities in terms of the health and safety of its population. The early diagnosis and identification of factors associated with overweight and obesity, especially in children and adolescents, are essential for the development of preventive measures to ensure the quality of life of the population in the future.

It is concerning that the prevalence of obese adolescents in our region is higher than that in other regions of Brazil, suggesting a need to analyze the underlying reasons and propose prevention measures. Therefore, factors such as ethnicity, pubertal stage, and socioeconomic status, among others indicated in the literature, must be examined in relation to the current increase in obesity among children and adolescents.

The present study did not observe any overall differences in the prevalence rates of overweight and obesity based on skin color; however, when grouped according to sex, we observed higher frequencies of obesity in boys (n=40, 62.5%) and overweight in girls (n=53, 55.7%) (*p*=0.035) among white individuals, which could be due to sedentary family lifestyles that favor the consumption of high-calorie foods.

An interesting finding was observed in the analysis of the prevalence of overweight and obesity according to pubertal stages. Males had abnormalities in the prepubertal period, while females had them later. It is assumed that in both sexes, in the pubertal period, adipose tissue tends to accumulate in order to be redistributed later during growth spurts. In the present study, we found that males not only accumulated more adipose tissue but also used more energy than the female group, resulting in a lower prevalence of overweight and obesity.

Few Brazilian studies have described the prevalence of overweight and obesity in relation to pubertal development and sexual maturation. In schools in Recife in the state of Pernambuco (PE) 2, overweight was more prevalent in girls who experienced early-onset puberty than in girls with normal pubertal development in an analysis of both male and female students. The prevalence rates of overweight and obesity increase in the final stages of sexual maturation; thus, each stage of pubertal development was considered separately, and individuals in early puberty were identified during the physical examinations that complemented the self-assessments 2. In a study in Florianópolis in the state of Santa Catarina (SC) 4 that involved students between the ages of 10 and 14 years, higher prevalence rates of overweight and obesity were identified in girls.

Excess weight has been linked to an individual's economic status in several studies. Overweight and obesity have been shown to be prevalent in populations belonging to different economic classes, and as in our sample, they have been observed more often in economic classes A+B. The 2008-2009 POF 7 showed an overall increase in food purchases, thus narrowing the gap between lower- and higher-income families. Students in the Vale do Jequitinhonha region belonging to families from the highest economic class (Class A) had a 2-fold higher risk of overweight and a 3-fold higher risk of obesity than their classmates belonging to families from the lowest economic class (Class E) 24. These results, as well as the results of the present study, indicate that the prevalence rates of overweight and obesity increase as the socioeconomic level increases. In contrast, in Ouro Preto (MG) 22, there were no significant differences among family income, parental education level, food intake, BMI and %BF.

Correlations were observed between parents or heads of households with higher education levels and normal body weight, given that parents with more education should be more aware of the importance of healthy eating habits and regular physical activity, which are required to maintain good health. In our study, significant differences in the prevalence of overweight and obesity were observed in participants whose heads of household had 12 or more years of formal education, with overweight most frequently observed among females n=70 (61.9%), and obesity most frequently observed among males n=48 (57.2%). These results contradict the expected benefits of higher education levels in terms of healthy habits and lifestyles. However, it could be assumed that because higher education levels are also related to higher socioeconomic statuses, there is greater exposure to inappropriate food choices and a greater ability to purchase high-calorie foods.

In Pelotas (RS) 25, there were increases in the percentages of overweight and obesity in children whose mothers had up to 12 years of education, and in the Vale do Jequitinhonha region 24, school children whose parents had 12 or more years of education were 1.5 times more likely to be overweight and 2 times more likely to be obese than children whose parents had completed less than 4 years of education. These results corroborate our findings; however, we did not analyze the odds of being overweight in terms of parental education levels. One review article indicated that there were increased prevalence rates of overweight and obesity in children of mothers with more than 4 years of education and more favorable socioeconomic living situations 26.

The prevalence of overweight and obesity has been described for different age groups in school populations in Brazil by several researchers. In this study, both public and private schools were included, and the overall prevalence rates of overweight and obesity in them were 17.3% and 15.0%, respectively. Furthermore, the prevalence rates of overweight and obesity were more significant in state public schools. Similar results were reported in the description of the prevalence rates of overweight and obesity in public and private schools in the city of Santos in the state of São Paulo (SP), with rates of 15.7% and 18.0%, respectively 27. Private school students had a 20% higher chance of being overweight than students from public schools in Maringá in the state of Paraná (PR) 28, and in Maceió in the state of Alagoas (AL) 3, students who attended private schools were twice as likely to be overweight and 5 times more likely to be obese. Among school children in the Northeast Region of Brazil 1, 54.5% of students in private schools had excess weight, including 42.8% who were overweight, whereas 15.6% students in public schools had excess weight, including 5.1% who were overweight.

In the present study, the time spent watching TV was added to the time spent playing video games and using the computer, and higher prevalence rates of overweight in females and obesity in males were observed when children spent more than 5 hrs/day on these activities.

Research on sedentary habits is scarce in the literature, especially in developing countries 29. However, a study of students from public and private schools in 26 state capitals and the Federal District 30 indicated that they spent a large amount of time watching TV, and this habit was associated with an increase in the consumption of high-calorie and sugary foods.

In an Egyptian study 31 describing the prevalence of overweight and obesity in adolescents between the ages of 14 and 19 years, the prevalence of excess weight was significantly higher when the time spent watching TV and/or using the computer was greater than 2 hours. In addition, physical inactivity and sedentary behavior, along with too much time spent watching TV and/or using the computer, were all associated with the risk of being overweight among the adolescents in the study.

In Ouro Preto (MG) 22, there was no association between excess body fat and physical and sedentary activities. In the 2009 National School-Based Health Survey (Pesquisa Nacional de Saúde do Escolar - PeNSE, 2009) 30, a statistically significant association was observed between the regular consumption of sweets, soft drinks, cookies and sausages and spending more than 2 hours watching TV on a daily basis. One explanation for the increased consumption of unhealthy foods is the amount of time in which children are exposed to commercials advertising these foods on TV. The food industry is the largest purchaser of commercial airtime on TV, and this vehicle is the simplest media source for food advertising. The industries that produce high-calorie foods and drinks aggressively advertise to teens in an attempt to build brand recognition/awareness, preference and loyalty 30,32.

In the city of Uberaba (MG), the prevalence of overweight was 17.3% (18.6% in females and 15.3% in males), and the prevalence of obesity was 15.0% (12.5% in females and 18.6% in males). These findings could reflect the reality of this disease in other similar Brazilian cities. The prevalence rates were significantly higher when the time spent watching TV and participating in other media activities was greater than 5 hrs/day, when individuals belonged to a higher economic class and when the head of the household had 12 or more years of education. It is important to emphasize the need to increase our understanding of the factors associated with this issue, and it is essential to implement measures and policies aimed at reversing this trend, such as stimulating healthy eating habits and physical activity and reducing the time children spend watching TV and participating in other media activities, including video games and social networking.

## AUTHOR CONTRIBUTIONS

Silva AP collected the data, conducted the data analysis and drafted the manuscript. Feilbelmann TC collected data and conducted the data analysis. Silva DC collected data and contributed to the final version of the manuscript. Palhares HM collected data and contributed to the final version of the manuscript. Scatena LM was involved in the sample calculation and assisted in the data analysis. Resende EA contributed to the project design and the final version of the manuscript. Borges MF guided the work, conducted the data analysis and contributed to the final version of of the manuscript.

## Figures and Tables

**Table 1:** Prevalence rates of overweight and obesity grouped according to demographic and socioeconomic variables.

Variables	N (Total)	Overweight n (%)	Obese n (%)	*p*
Sex				
Male	152	68 (44.7)	84 (55.3)b	0.004
Female	212	127 (59.9)a	85 (40.1)	
Ethnicity				
White	159	77 (48.4)	82 (51.6)	-
Non-white/non-black	155	88 (56.8)	67 (43.2)	-
Black	50	30 (60.0)	20 (40.0)	-
Stage of Puberty				
Prepubertal	128	63 (49.2)	65 (50.8)	-
Pubertal	121	59 (48.8)	62 (51.2)	-
Post-pubertal	115	73 (63.5)c	42 (36.5)d	0.036
Socioeconomic Status				
A+B	179	92 (51.4)	87 (48.6)	-
C	158	91 (57.6)	67 (42.4)	-
D+E	27	12 (44.4)	15 (55.6)	-
Education Level of the Head of the Family (Years of Study)				
<8	87	42 (48.3)	45 (51.7)	-
≥8 and <12	74	45 (60.8)	29 (39.2)	-
≥12	197	106 (53.8)	91 (46.2)	-
School Administrative Category				
Private	76	40 (52.6)	36 (47.4)	-
State public	194	107 (55.2)	87 (44.8)	-
Municipal public	94	48 (51.1)	46 (48.9)	-

a, b, c, d - *χ*^2^ test, level of significance *p*≤0.05.

**Table 2:** Prevalence rates of overweight and obesity grouped according to gender and demographic and socioeconomic variables.

Variables	Male			Female			M x F
	N	Overweight n (%)	Obese n (%)	N	Overweight n (%)	Obese n (%)	*p*
Ethnicity							
White	64	24 (37.5)	40^a^ (62.5)	95	53^b^ (55.7)	42 (44.3)	0.035
Non-white/non-black	66	33 (50.0)	33 (50.0)	89	55 (61.8%)	34 (38.2)	-
Black	33	22 (66.7)	11 (33.3)	28	19 (67.8)	9 (32.2)	-
Economic Class							
A+B	75	31 (41.3)	44^c^ (58.7)	104	61^d^ (58.6)	43 (41.4)	0.016
C	65	32 (49.2)	33 (50.8)	93	59 (63.4)	34 (36.6)	-
D+E	12	5 (41.6)	7 (58.4)	15	7 (46.7)	8 (53.3)	-
Education Level of Head of Family(Years of Study)							
<8	33	12 (36.4)	21 (63.6)	54	30 (55.5)	24 (44.5)	-
≥8 and <12	33	19 (57.6)	14 (42.4)	41	26 (63.4)	15 (36.6)	-
≥12	84	36 (42.8)	48^e^ (57.2)	113	70^f^ (61.9)	43 (38.1)	0.006
School Administrative Category							
Private	35	18 (51.4)	17 (48.6)	41	22 (53.6)	19 (46.4)	-
State public	60	31 (51.6)	49^g^ (48.4)	114	76^h^ (66.7)	38 (33.3)	<0.0001
Municipal public	37	19 (51.3)	18 (48.7)	57	29 (50.9)	28 (49.1)	-
Sedentary Leisure: TV, Computer, Video Games(Hours/Day)							
<2	15	7 (46.7)	8 (53.3)	26	14 (53.8)	12 (46.1)	-
≥2 and <5	72	33 (45.8)	39 (54.2)	92	52 (56.5)	40 (43.5)	-
≥5	50	23 (46)	27^i^ (54)	71	47^j^ (66.2)	24 (33.8)	0.027

a, b, c, d, e, f, g, h, i, j - *χ*^2^ test, level of significance *p*≤0.05.

**Table 3:** Correlations between %BF and time of sedentary activity in hours/week and between %BF and time of physical activity in minutes/week grouped by gender, according to the nutritional status of school children in Uberaba (MG).

Groups	N		Male		Female	
	M	F	R	*p*	R	*p*
%BF x Sedentary Activity						
Overweight	68	127	0.402	0.001	0.251	0.004
Obese	84	85	0.104	0.347	0.081	0.460
%BF x Physical Activity						
Overweight	68	127	-0.75	0.543	0.023	0.795
Obese	84	85	0.114	0.302	0.04	0.716

Spearman’s Correlation, level of significance *p*≤0.05.
